# Cellular Phenotypic Transformation During Atherosclerosis: The Potential Role of miRNAs as Biomarkers

**DOI:** 10.3390/ijms26052083

**Published:** 2025-02-27

**Authors:** Souhir Wassaifi, Bertrand Kaeffer, Sinda Zarrouk

**Affiliations:** 1 LR99E10 Human Genetics Laboratory, Faculty of Medicine of Tunis, University of Tunis El Manar, Tunis 1002, Tunisia; souhir.wassaifi@fst.utm.tn; 2 UMR 1280, PhAN, INRAE, Nantes Université, F-44000 Nantes, France; bertrand.kaeffer@univ-nantes.fr; 3 Institut Pasteur Tunis, University of Tunis El Manar, Tunis 1068, Tunisia

**Keywords:** microRNAs, arterial plaque rupture, plaque stability, heart attack

## Abstract

Cellular phenotypic transformation is a key process that occurs during the development and progression of atherosclerosis. Within the arterial wall, endothelial cells, vascular smooth muscle cells, and macrophages undergo phenotypic changes that contribute to the pathogenesis of atherosclerosis. miRNAs have emerged as potential biomarkers for cellular phenotypic changes during atherosclerosis. Monitoring miR-155-5p, miR-210-3p, and miR-126-3p or 5p levels could provide valuable insights into disease progression, risk of complications, and response to therapeutic interventions. Moreover, miR-92a-3p’s elevated levels in atherosclerotic plaques present opportunities for predicting disease progression and related complications. Baseline levels of miR-33a/b hold the potential for predicting responses to cholesterol-lowering therapies, such as statins, and the likelihood of dyslipidemia-related complications. Additionally, the assessment of miR-122-5p levels may offer insights into the efficacy of low-density-lipoprotein-lowering therapies. Understanding the specific miRNA-mediated regulatory mechanisms involved in cellular phenotypic transformations can provide valuable insights into the pathogenesis of atherosclerosis and potentially identify novel therapeutic targets.

## 1. Introduction

Atherosclerosis (AS), the main underlying cause of coronary artery disease (CAD), is a chronic, progressive inflammatory disease that results in the buildup of plaque inside the arteries [1]. The pathogenesis of atherosclerotic lesion formation is a multistep process involving immune and non-immune cellular phenotypic transformation of wall vessels [2]. The variable association of changes in the intima of the large and medium caliber arteries is accompanied by phenotypic changes in the media, preferentially at the level of the branching points of the large arteries and the internal curvature of the aortic arch, which are characterized by a disturbed blood flow. Endothelial cell (EC) dysfunction is initiated by upregulating inflammatory genes of adhesion molecules and chemokines [3].

Hypercholesterolemia also induces endothelial damage, allowing for the entry of lipids, such as low-density lipoprotein (LDL) particles, into the subendothelial space of the intimal layer of the arteries where they are further oxidized and act as a potent chemoattractant [4,5].

The inflamed endothelium recruits inflammatory monocytes to the arterial wall where they differentiate into macrophages. The latter polarizes into macrophages M1 (pro-inflammatory), M2 (anti-inflammatory), or Mox (oxidative stress phenotype) [6,7]. Macrophages further increase pro-inflammatory cytokines, which induce the recruitment and proliferation of vascular smooth muscle cells (VSMCs). The VSMC phenotype changes from the normal contractile type to the pathological proliferative type [8]. Monocyte-derived macrophages and macrophage-like VSMCs can internalize and accumulate oxidized LDL (ox-LDL), leading to the formation of foam cells, “foamy cells”, engaging inflammatory T and B cells in the expanding layer of the arterial intima [4,9,10,11].

The clinical evolution is towards the narrowing of the arterial lumen, plaque ulceration, and thrombosis. Advanced vulnerable plaques are rich in inflammatory cells, mainly “classically polarized” macrophages, and are very susceptible to rupture [12]. The characteristics of vulnerable plaques include thin fiber caps, large lipid cores, plaque inflammation, neovascularization in the plaques, intraplaque hemorrhage, and intraluminal thrombosis [3].

Rupture of the fibrous cap of the unstable plaque leads to platelet adhesion, thrombus formation, occlusion of arteries, and acute ischemic disorders [9]. Moreover, in more advanced plaques, EC-induced angiogenesis leads to new vessels invading the intima, a process closely related to plaque growth, destabilization, and rupture. Although there are many angiogenic inducers, vascular endothelial growth factor (VEGF) and basic fibroblast growth factor (bFGF) are probably the most critical and potent. The pro-angiogenic effect of VEGF and bFGF is mediated by VEGFR2, which is selectively expressed in vascular ECs, or FGF receptor 1 (FGFR1), respectively [13,14]. Activation of these receptors stimulates the angiogenic cascade, which leads to extracellular matrix degradation, migration of ECs to the perivascular space, proliferation, and tubal formation. Several microRNAs (miRNAs) modulate the function of ECs, VSMCs, and macrophages by controlling the expression levels of chemokines and, thereby, affecting different stages of atherosclerosis progression [15].

Previous reviews have extensively covered various aspects of miRNAs in atherosclerosis and CAD. For example, the regulation of atherosclerosis by miRNAs and their clinical implications have been discussed [16]. The role of miRNAs in diagnosing stable atherosclerosis across different arterial territories has been critically reviewed [17]. The pathogenesis of coronary artery disease through miRNA activity has also been explored [18]. Other reviews have focused on miRNAs as potential biomarkers and therapeutic targets in atherosclerosis [19,20]. Additionally, the expression profiles and impacts of miRNAs in advanced atherosclerotic plaques have been investigated [21,22]. Despite these comprehensive reviews, there is a need for an updated synthesis that specifically reassesses the effects of miRNAs on the molecular steps of the cellular phenotypic switch during atherosclerosis formation, with a focus on paving the way for new molecular therapeutics. This review aims to provide a deeper understanding of how miRNAs influence the key cellular transformations in atherosclerosis, offering insights that could lead to novel therapeutic strategies.

## 2. MicroRNAs in Cardiac Physiology: Biogenesis and Regulatory Mechanisms

**Figure 1:**
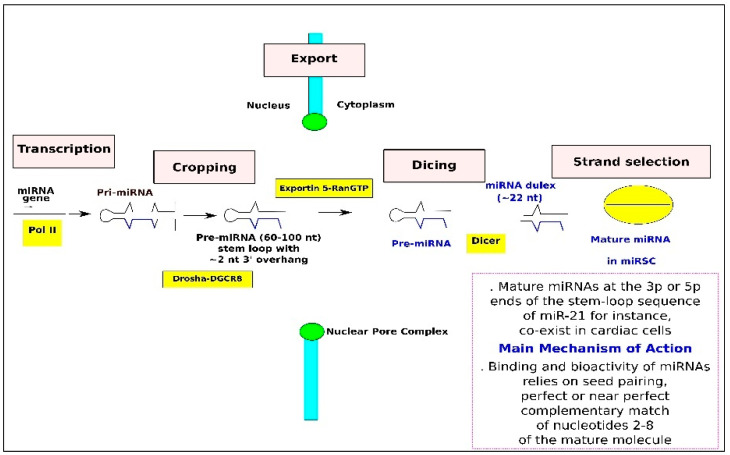
MicroRNA Biogenesis Pathway and main mechanism of action. MicroRNA (miRNA) genes are transcribed by RNA polymerase II (pol II), leading to pri-miRNAs (primary transcript). The «cropping» step is mediated by the Drosha–DGCR8 nuclear complex. The product of this nuclear processing step is a ~70-nucleotide (nt) pre-miRNA, which possesses a short stem plus a ~2-nucleotide 3′ overhang. This structure is recognized by the nuclear export factor exportin-5. Pre-miRNA constitutes a transport complex together with exportin-5 and its cofactor Ran (GTP form). Following export, the cytoplasmic RNase III Dicer participates in another processing step (‘dicing’) to produce miRNA duplexes. The duplex is separated, and usually, one strand is selected as the mature miRNA, whereas the other strand is degraded. However, both 3p and 5p ends of a specific stem-loop sequence (like miR-21-3p or 5p) can be produced in different cardiac cells or within the same cell, leading to potential clinical application [39]. The main mechanism of action is the binding of miRNAs to the 3′-untranslated region (UTR) of mRNA, referred to as seed pairing, the perfect or near perfect complementary match of nucleotides 2–8 of the mature miRNA. The illustration is adapted from Kim 2005 [40].

MicroRNAs (miRNAs) are short, single-stranded, non-coding RNAs, typically 19–25 nucleotides in length, highly conserved across evolution and predominantly located in non-coding regions of the genome. Despite not encoding proteins, they play pivotal roles in various physiological and pathological processes. By complementing and pairing with the 3′-UTR region of target gene mRNAs, miRNAs can regulate protein expression levels post-transcriptionally, impacting up to 30% of protein expression [23,24,25,26,27]. miRNAs are transcribed as primary miRNA (pri-miRNA) transcripts, which feature a characteristic hairpin structure with mature sequences at both the 5′ and 3′ ends [28]. These molecules can be expressed by the same cell or different cells within a tissue [29]. In cardiac diseases, the balance between 3p and 5p miRNA molecules may be linked to the effects of shear stress (SS) on endothelial evolution during atherosclerosis. Following recognition by RNA polymerase II (Pol II), miRNA promoters undergo splicing and polyadenylation in the nucleus [30,31]. Pri-mRNAs serve as substrates for the Drosha/DGCR8 complex, yielding pre-miRNAs [32]. Drosha cleaves pri-mRNA 11 bp from the recognition site, generating pre-miRNAs [33,34], which are subsequently exported from the nucleus to the cytoplasm via exportin 5 [35]. Within the cytoplasm, pre-miRNAs undergo processing by the Dicer/TRBP complex, resulting in miRNA duplexes. RNA helicases mediate chain selection, leading to the degradation of one strand, while the other matures into a functional miRNA. This mature miRNA then joins the RNA-induced silencing complex (RISC) [36], guiding the RISC to target mRNAs for degradation or translation inhibition [37]. Altered miRNA processing due to Dicer deletion can induce pro-inflammatory characteristics in vitro, leading to the upregulation of pro-inflammatory mediators in atherosclerotic lesions [38] (Figure 1).

## 3. Phenotypic Modulation of Endothelial Cell Phenotype by miRNAs and During Cell Recycling Through Apoptosis or Senescence

During AS, ECs undergo a phenotypic transformation characterized by an increased expression of adhesion molecules (vascular cell molecule (VCAM)-1, intercellular cell adhesion molecule (ICAM)-1, activated leukocyte cell adhesion molecule (ALCAM), and E-selectin), secretion of pro-inflammatory cytokines (tumor necrosis factor (TNF)-α), impaired production of nitric oxide (NO), as well as leukocyte infiltration [41]. This inflammatory process is induced by the decrease in endothelial SS, specifically low SS, which increases the supply, production, and oxidation of LDL in the fatty streaks of the arterial wall [42].

The inflammatory phenotype induced in ECs by variations in SS is regulated by several miRNAs acting in molecular networks. Both miR-92a-3p and miR-103-3p are upregulated in ECs in response to oxidized LDL (oxLDL), low SS, and oxidative stress. The miR-92a-3p cooperates with miR-103-3p to suppress Kruppel-like factor-4 (KLF4) and enhance inflammatory gene expression through the activation of nuclear factor-kappa B (NF-κB) [43,44,45]. Additionally, miR-92a-3p targets several other transcripts in ECs, such as Kruppel-like factor-2 (KLF2), suppressor of cytokine signaling 5 (SOCS5), and Sirtuin-1 (SIRT1) [43,46]. These transcripts positively regulate endothelial nitric oxide synthase (eNOS)-derived NO in arterial endothelium and contribute to the inflammatory phenotype. Hence, inhibiting the interaction between miR-103-3p and KLF4 with a sequence-specific oligonucleotide ameliorates atherosclerosis [45]. The direct inhibition of miR-103-3p also reduces atherosclerosis, endothelial inflammation, and endoplasmic reticulum stress by targeting the protein phosphatase and tensin homolog (PTEN) [47].

Oscillatory shear stress (OSS), a form of low SS, also induces monocyte adhesion to ECs by upregulating miR-663.This upregulation is associated with altered expression of several transcription factors, including KLF4, CCAAT-enhancer-binding protein beta (C/EBPB), and activating transcription factor 3 (ATF3) [48]. Moreover, in in vitro studies using human umbilical vein endothelial cells (HUVEC), low SS leads to sustained expression of miR-21-5p. This miRNA promotes increased activity of activator protein-1 (AP-1) by targeting peroxisome proliferator-activated receptor alpha (PPAR-α), thereby enhancing the expression of VCAM-1 and monocyte chemotactic protein-1 (MCP-1), ultimately facilitating the adhesion of monocytes to ECs [49,50]. Furthermore, in response to SS, miR-10a-5p is downregulated in atherosusceptible areas, as observed in in vivo studies using artery site-specific profiling in normal adult swine. This downregulation fosters IκB/NF-κB-mediated inflammation through repression of mitogen-activated protein kinase kinase kinase 7 (MAP3K7) and β-transducin repeat containing (β-TRC) [51,52].

miR-155-5p has been shown to increase EC response to laminar flow (l-flow) and bind to both anti-inflammatory targets, such as eNOS [53,54], and pro-inflammatory targets, such as myosin light chain kinase (MLCK) and type I receptor angiotensin II (AT1) [55,56]. Furthermore, in vitro studies have shown that overexpression of miR-34a-5p in ECs exacerbates endothelial dysfunction, inflammation, and vascular injury by affecting NF-κB-mediated expression of ICAM-1 and VCAM-1 through targeting SIRT1 under OSS, while it reversibly affects these expressions under high SS (HSS) [57]. miR-221/222 also targets ICAM-1 in ECs, and its expression is repressed by the HIV transactivator of transcription (Tat) through an NF-κB-dependent mechanism, leading to a decrease in ICAM-1 levels and an increase in monocyte endothelial adhesion [58]. Moreover, in vitro studies have demonstrated that upregulation of miR-221/222 effectively reduces the inflammatory response induced by AII in ECs by targeting V-ets erythroblastosis virus E26 oncogene homolog 1 (Ets-1) and its downstream genes, including VCAM-1 and MCP-1 [53]. Furthermore, miR-31-5p and miR-17-3p have been identified as regulators of TNF-induced E-selectin and ICAM-1 expression, operating in a negative feedback loop to modulate EC activation, as reported by Suarez Y et al. [59]. TNF induces endothelial dysfunction by decreasing eNOS expression in the endothelium through the NF-kB-dependent biogenesis of miR-31-5p [60]. Additionally, TNF promotes the upregulation of miR-141-3p, an miRNA that targets ICAM-1 and reduces the adherence of leukocytes to endothelial surfaces [61]. Moreover, miR-223-3p targets ICAM-1, and its repression by HDL-transferred miR-223-3p may contribute to EC dysfunction in vitro [62,63]. Furthermore, miR-181a-5p and miR-181a-3p are downregulated under conditions of hyperlipidemia and coronary artery disease. When upregulated, these miRNAs reduce endothelial inflammation via NF-kB pathway modulation, decreasing leukocyte-endothelial adhesion by targeting VCAM-1, ICAM-1, and E-selectin genes. These effects have been demonstrated both in vitro using HUVECs and in vivo in apoE−/− mice models [64]. In contrast, let-7g-5p exerts anti-inflammatory effects on ECs when upregulated by inhibiting SIRT1 and transforming growth factor (TGF)-β signaling [65].

In atherosclerotic lesions, apoptotic ECs release apoptotic bodies enriched in miR-126-3p, which have been found to have a protective effect in mice. This effect is achieved by targeting the regulator of G protein signaling 16 (RGS16), a negative regulator of the C-X-C motif chemokine receptor 4 (CXCR4), and by promoting CXCL12-dependent recruitment of progenitor cells to the endothelial lining [66,67,68]. Moreover, overexpression of miR-126-3p inhibits apoptosis in vascular ECs (VECs) by targeting the antiapoptotic phosphoinositide 3-kinase/protein kinase B (PI3K/Akt) pathway via phosphoinositide-3-kinase regulatory subunit 2 (PIK3R2) [69]. In parallel, miR-126-5p is upregulated in ECs, where it reduces vascular inflammation by targeting and downregulating VCAM-1, ALCAM, and SET domain containing 5 (SetD5). This regulatory role has been demonstrated through both in vitro studies using cultured endothelial cells and in vivo studies in living organisms [70,71]. Additionally, low SS inhibits miR-126-5p and promotes lesion development by activating the inhibitor of EC proliferation delta-like 1 homolog (Dlk1) [72].

During apoptosis, both miR-21-5p and miR-21-3p were found to have binding sites in the 3′ untranslated region (UTR) of PTEN [39]. Recent studies have revealed that miR-217-5p, upregulated in ECs, targets chloride intracellular channel 4 (CLIC4) to protect ECs from death induced by ox-LDL in atherosclerosis, as evidenced by in vitro studies using cultured ECs treated with ox-LDL [73]. Similarly, miR-151-3p, downregulated by ox-LDL, targets interleukin-17A (IL-17A) and inhibits the apoptosis of ECs induced by ox-LDL in atherosclerosis. These effects were shown in vitro using human aortic endothelial cells (HAECs) [74]. Likewise, miR-495-3p, which is downregulated in patients with coronary artery disease, targets chemokine (C-C motif) ligand 2 (CCL2) and reduces apoptosis by affecting the cleaved caspase-3 production in HUVECs, as demonstrated in vitro [75]. On the contrary, miR-132-3p upregulation promotes apoptosis in HUVECs by inhibiting SIRT1 [76,77]. According to Zhang et al., the upregulation of miR-30 by a high-fat diet (HFD) inhibits the protein translation of autophagy-associated protein 6 (ATG6), which may compromise the protective benefits of EC autophagy against atherosclerosis in ApoE (−/−) mice [78]. On the other hand, upregulation of miR-210-3p was found to have a proatherosclerotic role in the development of atherosclerosis by targeting 3-phosphoinositide-dependent protein kinase-1 (PDK1) and blocking the P13K/Akt/mTOR signaling pathway, leading to increased EC apoptosis in a mouse model of atherosclerosis induced by a high-fat diet (HFD) [79]. miR-142-3p overexpression encourages EC apoptosis by inhibiting the downstream target rapamycin-insensitive partner of the mammalian target of rapamycin (Rictor) and the Akt/eNOS signaling pathway [80]. Furthermore, upregulation of miR-122-5p has been proposed to have a pro-apoptotic function in vitro using HAECs by specifically targeting the X-linked inhibitor-of-apoptosis protein (XIAP) [81]. Downregulation of miR-17-5p inhibits HUVEC apoptosis via the extracellular signal-regulated kinase (ERK) pathway [82]. Upregulation of miR-365 and miR-34a-5p directly targets the antiapoptotic B cell lymphoma 2 (Bcl-2) and promotes cell death in both in vitro and in vivo studies [83,84][. miR106a-5p participates in oxLDL-stimulated apoptosis and oxidative injury in HUVECs by regulating the signal transducer and activator of transcription 3 (STAT3) [85]. It was discovered that miR-26a-5p, involved in endothelial apoptosis, is downregulated in atherosclerosis and directly targets transient receptor potential cation channel subfamily C member 6 (TRPC6) in VEC [86].

Furthermore, certain miRNAs play crucial roles in senescence pathways. For instance, upregulation of miR-217-5p, miR-21-5p, and miR-34a-5p contributes to EC senescence promotion, whereas downregulation of let-7g counters this effect by targeting the SIRT1 gene [65,87,88,89]. Similarly, upregulation of miR-10a-3p, miR-21-5p, and miR-22-5p contributes to the senescence of endothelial progenitor cells (EPCs) by inhibiting the expression of specific genes, such as high-mobility group A2 (HMGA2) and AKT3 [90,91]. Moreover, miR-146a upregulation retards EC senescence by targeting NADPH oxidase 4 (NOX4), studied in HUVECs [92], while the upregulation of miR-20b enhances cell survival and prevents senescence in HUVECs by modulating the Wnt/β-catenin pathway through the thioredoxin interacting protein/NOD-like receptor family pyrin domain containing 3 (TXNIP/NLRP3) axis [93]. Recent studies also suggest that miR-30a-5p overexpression and the inhibition of miR-30a-3p and miR-181a-5p induce senescence [94,95]. The specific roles of miRNAs in these processes, including their modulation of endothelial cell phenotype during atherosclerosis, are detailed further in Table 1.

## 4. Modulation of Macrophage Plasticity and Polarization

Macrophage polarization plays a crucial role in atherosclerosis, involving the transition between pro-inflammatory M1 and anti-inflammatory M2 phenotypes [96]. Experiments have revealed miRNAs uniquely regulated in human macrophages polarized toward M1 (miR-125a-3p and miR-26a-3p) and M2 (miR-193b, miR-27a-3p, miR-29b-3p, miR-132-3p, and miR-222-3p). The increased expression of miR-155-3p indicates its involvement in both M1 and M2 polarization, previously associated with levels of post lipopolysaccharide (LPS) responsiveness. Specifically, miR-125a-3p, -193b, miR-27a-3p, miR-155-3p, and miR-29b-3p exhibit increased expression during macrophage activation, regardless of M1 or M2 phenotype, while miR-26a shows decreased expression. miR-222-3p expression is paradoxically upregulated in M2 macrophages but downregulated in M1 macrophages [97]. Comparatively, Zhang et al. observed distinct miRNA expression profiles in mice with M1 versus M2 macrophages, with miR-181a, miR-155-5p, miR-204-5p, and miR-451 being highly upregulated and miR-125-5p, miR-146a, miR-143-3p, and miR-145-5p significantly downregulated [98]. Furthermore, miR-223-3p emerges as a pivotal regulator of macrophage polarization. It functions by inhibiting the Pknox1 protein, thereby impeding the activation of pro-inflammatory macrophages [99]. Additionally, upregulation of miR-30b-5p acetylates HMGB1 through the UBE2D2/KAT2B/HMGB1 pathway, thereby promoting the polarization of pro-inflammatory cells and facilitating macrophage recruitment in an in vitro cell culture study using HAEC [100]. Moreover, miR-146a-5p is upregulated in response to LPS-induced Toll-like receptor (TLR) activation and pro-inflammatory cytokines via the NF-B signaling pathway. This miRNA also exerts anti-inflammatory effects by inhibiting IL-1-induced activation through the suppression of TRAF6 and IRAK1 [101,102,103,104].

miR-21-5p overexpression modulates NF-kB signaling during TLR-mediated activation of human peripheral blood mononuclear cells by targeting programmed cell death protein 4 (PDCD4), thereby mitigating inflammation (Table 2). Conversely, downregulation of miR-21 leads to increased levels of TNFα and IL6 while decreasing IL10 LPS stimulation, suggesting an anti-inflammatory role [105,106]. As shown Table 2, miR-155-5p promotes inflammatory responses by targeting BCL6, SOCS-1, and Src homology 2 domain-containing inositol polyphosphate 5-phosphatase 1 (SHIP1) [104,107,108,109] while also potentially exhibiting an anti-inflammatory effect through targeting MAP3K10 [110]. Moreover, upregulation of miR-342-5p enhances the expression of pro-inflammatory macrophage mediators, including inducible eNOS and IL6, by suppressing Akt1-mediated inhibition of miR-155 in early atherosclerotic lesions in Apoe (−/−) mice [111]. Inhibition of miR-125a-5p correlates with elevated levels of inflammatory cytokines, such as TGFβ, TNFα, IL2, and IL6, leading to inflammation reduction [112]. Conversely, upregulation of miR-125b potentiates pro-inflammatory macrophage activation by targeting IFN regulatory factor 4 (IRF4) in both in vitro cell culture and in vivo mouse models [113] (Table 2).

## 5. Foam Cell Formation and Cholesterol Efflux Under MiRNA Regulation

miRNAs are pivotal regulators in the formation of foam cells, intricately modulating various steps involved in cholesterol and fatty acid metabolism. For instance, upregulation of miR-125a-5p and miR-146a-5p have been demonstrated to attenuate lipid uptake and cytokine release in oxLDL-stimulated macrophages, partly through the targeting of oxysterol binding protein-like genes 9 (ORP9) and TLR4, respectively, suggesting a potential protective role in atherosclerosis development [112,114]. Conversely, inhibition of miR-146b-5p exacerbates the inflammatory response and enhances lipid uptake during foam cell formation by targeting TRAF6 [115]. Dicer, a key enzyme in miRNA biogenesis, exerts a critical role in enhancing mitochondrial oxidative metabolism during alternative macrophage activation and foam cell formation by suppressing nuclear receptor corepressor (Lcor) through miR-10a-5p, thereby safeguarding macrophages against lipid overloading [38].

Furthermore, upregulation of miR-155-5p has been implicated in augmenting oxLDL-induced foam cell formation by targeting HMG box transcription protein 1 (HBP1), cluster differentiation 36 (CD36), Vav guanine nucleotide exchange factor 3 (Vav3), and SOCS1 genes [116,117]. Conversely, reducing miR-155-5p levels through CTRP12 leads to increased expression of liver X receptor α (LXRα), promoting ATP-binding cassette transporter A1 (ABCA1)- and ATP-binding cassette sub-family G member 1 (ABCG1)-dependent cholesterol efflux [118]. Additionally, inhibition of miR-200b-3p alleviates lipid accumulation and enhances cholesterol efflux by targeting ABCA1 in macrophage-derived foam cells [119]. Several other miRNAs, including miR-33-5p, miR-144-3p, miR-302a-3p, miR-23a-5p, miR-26a, miR-27a/b-3p, and miR-10b are also implicated in lipid metabolism and regulate cholesterol efflux pathways in macrophages, thereby influencing foam cell formation and inflammation in atherosclerosis by targeting ABCA1 [120,121,122,123,124,125,126,127]. Moreover, coenzyme Q10 has been shown to inhibit AP-1 and reduce miR-378 expression, consequently increasing ABCG1 expression and facilitating cholesterol efflux from macrophages both in vitro and in vivo [128]. These data are summarized in Table 3.

## 6. Phenotypic Modulation of Vascular Smooth Muscle Cells (VSMCs)

VSMCs undergo phenotypic modulation in atherosclerosis, transitioning from a contractile to a synthetic phenotype characterized by increased proliferation, migration, extracellular matrix production, and secretion of pro-inflammatory mediators. Upregulation of miR-221/222 contributes to the phenotypic modulation of VSMCs by targeting the cyclin-dependent kinase inhibitors p27Kip1 and p57Kip2, which are crucial regulators of cell cycle progression and apoptosis [129,130]. Similarly, upregulation of miR-21-3p enhances VSMC migration and proliferation in vitro by targeting PTEN [131], while overexpression of miR-663 increases the expression of VSMC differentiation marker genes and potentially inhibits PDGF-induced VSMC proliferation and migration by targeting JUNB, MYL9, and MMPs, thereby limiting their contractile ability [132]. Conversely, the upregulated miR-143/145 cluster maintains the contractile phenotype of VSMCs by targeting myocardin and the transcription factor NK2 homeobox 5 (Nkx2-5) [133,134]. Additionally, myocardin inhibits VSMC migration through the induction of miR-24 and miR-29a, subsequently inhibiting PDGF-b [135], and miRNA-24-3p is suggested to regulate the viability and apoptosis of VSMCs by targeting Bcl-2-like protein 11 (Bcl-2L11) [136]. Other miRNAs, such as miR-638 targeting nuclear receptor subfamily 4 group A member 1 (NOR1) [137] and Rho-associated coiled-coil kinase 2 (ROCK2) [138], miR-145-5p targeting Smad4 to disrupt the TGF-β signaling cascade [139], and overexpressed miR-532-5p targeting PDCD4 [140], suppress VSMC proliferation and migration. Furthermore, upregulation of miR-324-3p and downregulation of let-7e-5p contribute to vascular calcification (VC) by targeting insulin-like growth factor 1 receptor (IGF1R), phosphatidylinositol-4,5-bisphosphate 3-kinase catalytic subunit alpha (PIK3CA), and MAP2K1 in a cellular calcification model using the mouse VSMC line MOVAS-1 [141], underscoring the regulatory role of miRNAs in VSMC phenotypic modulation and vascular pathologies (Table 4).

## 7. Conclusions and Perspective for Using miRNAs in the Prediction of Atherosclerotic Plaque Rupture

**Figure 2:**
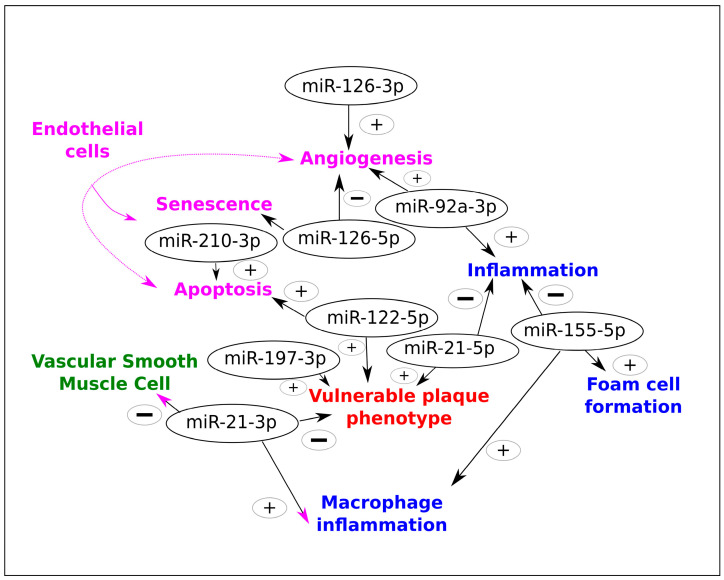
Main miRNAs involved in cellular phenotypic transformation with potential interest as biomarkers. The vulnerable plaque phenotype is at the crossroads between the regulation of miR-197-3p, miR-122-5p, 3p, and 5p forms of miR-21. The pri-mir-17-92 region is coding for six different miRNAs: miR-17, -18a, -19a, -20a, -19b, and -92a-3p. The mature form of miR-92a-3p is crucial in acquiring the atherosclerosis phenotype. The magenta color indicates that angiogenesis, apoptosis, or senescence is related to endothelial cell biology. Not presented in the figure, the miR-33a and b have been linked to cholesterol-lowering therapies. Signs (+) indicate miRNAs with proatherosclerotic function, while (−) indicates miRNAs with antiatherosclerotic function. The miR-33 gene family encompasses 2 forms in humans: one on chromosome 22 called miR-33a and one on chromosome 17 called 33b; both these non-coding genes produce 3p and 5p mature forms.

Recent advancements in understanding the role of miRNAs in atherosclerosis have provided promising perspectives for their utilization as predictive biomarkers in assessing plaque vulnerability and complications [142]. Baseline levels of miR-33a/b hold potential for predicting responses to cholesterol-lowering therapies, such as statins, and the likelihood of dyslipidemia-related complications [143,144]. Additionally, assessment of miR-122-5p levels may offer insights into the efficacy of LDL-lowering therapies and miR-122-5p inhibitors [81,145,146]. Elevated levels of miR-197-3p have shown associations with plaque vulnerability and increased cardiovascular risks, suggesting its potential as a predictive biomarker for assessing complications in atherosclerosis [147]. Similarly, monitoring miR-155, miR-210, and miR-126 levels could provide valuable insights into disease progression, risk of complications, and response to therapeutic interventions [116,117,148,149]. Moreover, miR-92a’s elevated levels in atherosclerotic plaques present opportunities for predicting disease progression and related complications [150] (Figure 2). These findings underscore the promising role of miRNAs as predictive biomarkers in improving risk stratification and guiding personalized therapeutic approaches in atherosclerosis. With Wassaifi’s thesis aiming to enhance clinical practices, the integration of these miRNA biomarkers into routine assessments could revolutionize risk assessment and treatment strategies [151], leading to more targeted and effective interventions tailored to individual patient profiles.

## Figures and Tables

**Table 1:** Function of microRNAs in endothelial cells in atherosclerosis.

Cell Origin	Antiatherosclerotic	Functions	Phenotype	Targets	Reference
Endothelial cell	Proatherosclerotic Inflammatory	Proatherosclerotic Inflammatory	Induces endothelial dysfunction	KLF2 KLF4 SIRT1 SOCS5 eNOS	[43,44,46]
	Antiapoptotic	Proatherosclerotic Inflammatory	Inflammatory response	KLF4	[45]
	Proatherosclerotic	Antiatherosclerotic	Inhibit inflammatory response	PTEN	[47]
	Antiatherosclerotic	Proatherosclerotic Inflammatory	Inflammatory response	IL8 ATF3 KLF4 C/EBPB	[48]
	Antiatherosclerotic Anti-inflammatory	Antiapoptotic	PTEN	PTEN	[39]
	Antiatherosclerotic	Proatherosclerotic	Neointimal lesion formation Inflammation Pro-oxidative stress effect Cell senescence	PPARα AP1 VCAM-1 MCP-1 SIRT1	[49,50,88]
	Proatherosclerotic	Antiatherosclerotic	Inhibit cell senescence	HMGA2	[90]
	Antiatherosclerotic	Antiatherosclerotic Anti-inflammatory	Lower levels in atherosusceptible regions Inhibits NF-κB activation	MAP3K7 β-TRC	[51,52]
	Proatherosclerotic	Antiatherosclerotic	Inhibit cell senescence	HMGA2	[90]
	Antiatherosclerotic	Proatherosclerotic	Inflammatory response Cell senescence and apoptosis	SIRT1 VCAM-1 ICAM-1 Bcl-2	[57,84,89]
	Antiatherosclerotic	Antiatherosclerotic	Inhibit inflammatory response	eNOS	[53,54]
	Proatherosclerotic	Proatherosclerotic	Enhances inflammation, promotes atherosclerosis	MLCK AT-1	[55,56]
	Antiatherosclerotic	Antiatherosclerotic	Inhibit inflammatory	ICAM-1 Ets-1 VCAM-1 MCP)-1	[58,59]
	Antiatherosclerotic	Antiatherosclerotic	Negative feedback control of inflammation	ICAM E selectine	[59]
	Anti-inflammatory	Proatherosclerotic	Endothelial dysfunction	eNOS	[60]
	Antiatherosclerotic Anti-inflammatory	Antiatherosclerotic	Inhibit endothelial adhesion of leukocytes	ICAM-1	[61]
	Antiapoptotic	Antiatherosclerotic	Inhibit endothelial adhesion of leukocytes	ICAM-1	[62,63]
	Antiatherosclerotic Anti-inflammatory	Anti-inflammatory	Suppressor of endothelial inflammatory responses	ICAM-1 VCAM-1	[64]
	Antiapoptotic	Antiatherosclerotic Anti-inflammatory	Inhibitory effect on the senescence of endothelial cells	SIRT1 (TGF)-b	[65]
	Proatherosclerotic	Antiapoptotic	Inhibits EC apoptosis	RGS16 CXCR4 CXCL12 PI3K/Akt	[66,67,68,69]
	Antiapoptotic	Antiatherosclerotic Anti-inflammatory	Inhibits angiogenesis and inflammation	VCAM-1 ALCAM SetD5 Dlk1	[70,71,72]
	Antiatherosclerotic Antiapoptotic	Antiapoptotic	Inhibited apoptosis of endothelial cells	CLIC4	[73]
	Proatherosclerotic	Proatherosclerotic	Cell senescence	SIRT1	[87,88]
	Proatherosclerotic	Antiapoptotic	Inhibited apoptosis of endothelial cells	IL-17A	[74]
	Pro-apoptotic	Antiatherosclerotic Antiapoptotic	Inhibition of EC apoptosis	CCL2	[75]
	Apoptotic	Proatherosclerotic	Promotion of EC apoptosis	SIRT1	[76,77]
	Apoptotic	Proatherosclerotic	Impairment of protective effects of endothelial cell autophagy	ATG6	[78]
	Antiapoptotic	Pro-apoptotic	Promoted EC apoptosis	PDK1 P13K/Akt/mTOR	[79]
	Proatherosclerotic	Apoptotic	Promoted EC apoptosis	Rictor Akt/eNOS	[80]
	Apoptotic	Apoptotic	Promoted EC apoptosis	XIAP	[81]
	Antiapoptotic	Antiapoptotic	Prevents endothelial cell apoptosis	ERK	[82]
	Antiatherosclerotic	Proatherosclerotic	Endothelial cell apoptosis	Bcl-2	[83]
	Antiatherosclerotic Anti-inflammatory	Apoptotic	Promoted EC apoptosis	STAT3	[85]
	Antiatherosclerotic	Antiapoptotic	Prevents endothelial cell apoptosis	TRPC6	[86]
	Antiatherosclerotic	Antiatherosclerotic	Inhibit cell senescence	AKT3	[91]
	Proatherosclerotic Inflammatory	Antiatherosclerotic Anti-inflammatory	Inhibit cell senescence Anti-inflammatory	NOX4	[92]
	Antiapoptotic	Antiatherosclerotic	Inhibits cell senescence	TXNIP/NLRP3	[93]

**Table 2:** Function of microRNAs in macrophages in atherosclerosis.

Cell Origin	MicroRNA	Functions	Phenotype	Targets	Reference
Macrophages					
	miR-223-3p	Antiatherosclerotic	Suppressing the pro-inflammatory activation of macrophages	Pknox1	[99]
	miR-30b-5p	Proatherosclerosis	Pro-inflammatory activation of macrophages	HMGB1	[100]
	miR-146a-5p	Anti-inflammatory	Reduction in inflammation	TRAF6 IRAK IL1	[101,102,103,104]
	miR-21-5p	Anti-inflammatory	Reduction in inflammation	PDCD4	[105,106]
	miR-155-5p	Proatherosclerosic	Pro-inflammatory activation of macrophages	SOCS-1 SHIP1 Bcl6	[104,107,108,109]
		Antiatherosclerotic	Inhibit inflammatory response	MAP3K10	[110]
	miR-342-5p	Proatherosclerotic	Induces pro-inflammatory mediators (NOS2, IL-6), promotes atherosclerosis	Akt1	[111]
	miR-125a-5p	Antiatherosclerotic	Anti-inflammatory activation of macrophages	TGF-β TNF-α IL-2 IL-2	[112]
	miR-125b	Pro-inflammatory	Pro-inflammatory activation of macrophages	IRF4	[113]

**Table 3:** Function of microRNAs in foam cell formation in atherosclerosis.

Cell Origin	MicroRNA	Functions	Phenotype	Targets	Reference
Foam cell and cholesterol efflux	miR-125a-5p	Antiatherosclerotic	Reduction in intracellular lipid accumulation Foam cell formation	ORP9	[112]
	miR-146a-5p	Antiatherosclerotic	Reduction in intracellular lipid accumulation Foam cell formation	TLR4	[114]
	mir-146b-5p	Antiatherosclerotic	Reduction in inflammation and intracellular lipid accumulation	TRAF6	[115]
	miR-10a-5p	Antiatherosclerotic	Reduction in intracellular lipid accumulation	Lcor	[38]
	miR-155-5p	Proatherosclerotic	Promotes foam cell formation	HBP1 CD36 Vav3 SOCS-1	[116,117]
		Antiatherosclerotic	Promotes cholesterol efflux	LXRα ABCA1 ABCG1	[118]
	miR-200b-3p	Antiatherosclerotic	Reduction in intracellular lipid accumulation and promotes cholesterol efflux	ABCA1	[119]
	miR-33-5p	Proatherosclerotic	Inhibits cholesterol export	ABCA1	[120,121]
	miR-144-3p	Proatherosclerotic	Promotes intracellular lipid accumulation and inhibits cholesterol export	ABCA1	[122]
	miR-302a-3p	Proatherosclerotic	Inhibits cholesterol export	ABCA1	[123]
	miR-23a-5p	Proatherosclerotic	Promotes foam cell formation and inhibits cholesterol export	ABCA1	[124]
	miR-26	Proatherosclerotic	Inhibits cholesterol export	ABCG1 ARL7	[125]
	miR-27a/b-3p	Proatherosclerotic	Inhibits cholesterol export	ABCG1 LPL	[126]
	miR-10b	Proatherosclerotic	Inhibits cholesterol export	ABCA1	[127]
	miR-378	Antiatherosclerotic	Promotes cholesterol export	ABCG1 AP-1	[128]

**Table 4:** Function of microRNAs in VSMC in atherosclerosis.

Cell Origin	MicroRNA	Functions	Phenotype	Targets	Reference
VSMC	miR-221/222	Proatherosclerotic	VSMC proliferation	c-Kit p27Kip1	[129,130]
	miR-21-3p	Proatherosclerotic	VSMC proliferation	PTEN	[131]
	miR-663	Antiatherosclerotic	Regulate VSMC phenotype switch	JUNB MYL9 MMPs	[132]
	miR-145/-143	Antiatherosclerotic	Regulate VSMC phenotype switch	myocardin Nkx2-5	[133,134]
	miR-24 miR-29	Antiatherosclerotic	Inhibits VSMC migration	PDGF-b	[135]
	miR-24-3p	Antiatherosclerotic	Inhibits VSMC proliferation and promotes VSMC apoptosis	Bcl-2L11	[136]
	miR-638	Antiatherosclerotic	Inhibit VSMC proliferation and migration	NOR1 ROCK2	[137,138]
	miR-145-5p	Antiatherosclerotic	Inhibit VSMC proliferation and migration	TGF-β Smad4	[139]
	miR-532-5p	Antiatherosclerotic	Inhibit VSMC proliferation and migration	PDCD4	[140]
	miR-324-3p	Proatherosclerotic	Vascular calcification	IGF1R PIK3CA MAP2K1	[141]
	Let-7e-5p	Proatherosclerotic	Vascular calcification	IGF1R PIK3CA MAP2K1	[141]
